# Agency-driven and community-driven impact in livelihood recovery: Beneficiaries stories

**DOI:** 10.4102/jamba.v14i1.1200

**Published:** 2022-10-14

**Authors:** Eko T. Paripurno, Gandar Mahojwala, Galih Prabaswara, Sabrina U. Khabibah

**Affiliations:** 1 University of Pembangunan Nasional Veteran Yogyakarta, Yogyakarta, Indonesia; 2 Disaster Management Centre, University of Pembangunan Nasional Veteran Yogyakarta, Yogyakarta, Indonesia

**Keywords:** disaster management, disaster recovery, community-driven approach, agency-driven approach, participatory rural appraisal

## Abstract

The effectiveness of agency-driven and community-driven recovery approaches is a discoursive material that appeals to many disaster management practitioners, scholars, international donors or governments. Meanwhile, the community has never been part of this discourse as beneficiaries to obtain its perspective on both approaches. Loli Dondo village, which received agency-driven and community-driven livelihood recovery after the 2018 Central Sulawesi Tsunami, was purposively selected as the site of investigation. This study aims to explore how both the approaches were perceived by the community. A qualitative research method involving focus group interviews (FGI), interviews and participatory rural appraisal (PRA) was used to explore and capture community narratives and collective storytelling to produce a visual analysis of the community’s problem-solving strategy and management. Based on the results, the community-driven recovery approach fulfilled the community standards and provided more impact than its counterpart. Several essential viewpoints were underlined for all approaches as follows: (1) the agency’s transparency to the community about financial conditions, (2) communication and active community involvement and (3) preciseness of the aid to community needs. These elements are expected to improve the implementation of such recovery programmes, thereby increasing quality and accessibility. The insightful community storylines, experiences and reflection support in building a better practice of livelihood recovery approaches for beneficiaries and also contribute widely to their implementation according to the community’s perspective.

**Contribution:**

These findings highlight the key elements of recovery implementation based on community perspectives to achieve impact of livelihood recovery.

## Introduction

On 28 September 2018, at 18:02 Central Indonesian Time, an earthquake occurred in Central Sulawesi with a magnitude of 7.4, followed by a tsunami that hit the west coast of Sulawesi Island. Donggala Regency was not as badly hit by the tsunami as Palu City; however, the impact caused difficulties for the residents, specifically people living close to the coast. Loli Dondo Village’s territory is the largest in Banawa Subdistrict, Donggala Regency, Central Sulawesi Province, yet it has the lowest population density with 101 inhabitants per km^2^ and a total population of 1356 (Indonesian Bureau of Statistics 2020). This landscape of the village contains many houses built on the Trans-Sulawesi Highway sides and the elongated hill located on the highway’s west side.

During the 2018 tsunami, houses closest to the beach were mostly destroyed; meanwhile, those near the hillside had moderate damage. Loli Dondo village’s land is majorly used for settlement and fishery; hence, the housing and public facilities experienced the tsunami’s greatest impact, such as mosques and fish markets. (Widiyanto et al. 2019). According to eyewitnesses, the first wave in this village arrived from Palu City’s direction approximately 4 min after the earthquake (Aránguiz et al. 2020), and another source reported 5 min (Stolle et al. 2020). Loli Dondo and other villages in Banawa Subdistrict lack an early warning system, but only four villages have evacuation route signs (Indonesian Bureau of Statistics 2020).

The agency-driven approach is considered the easiest and quickest way to return places affected by disaster to normality and protect communities containing high numbers of vulnerable individuals. This proved to be undesirable to the beneficiaries, because it seldom involves the target community’s active participation. Meanwhile, the community-driven approach has the advantage of empowering beneficiaries with less money compared to its counterpart, but a longer duration is required because of the participation process.

This study aims to explore the reflection of the community as beneficiaries in Loli Dondo village on agency-driven and community-driven approaches, which have not been practiced previously. Therefore, the community’s authentic reflection on identifying the characteristics and benefits of each approach tends to be obtained. The exploration is important because several sources state that approaches determine recovery effectiveness (Chang et al. 2010; Cretney 2018; Elkahlout 2020; Santiago et al. 2018; Sullivan & Sagala 2020). Participatory rural appraisal (PRA) usage also enriches the obtained data qualitatively, by presenting visual analysis that was conducted in a participative manner with the community. Fishermen organisations as beneficiaries are the participants selected. Furthermore, the study provides a significant impact on post-disaster recovery approaches. This concerns increasing understanding of how each approach can contribute to disaster recovery and the means of adapting to each approach and supporting the community-driven process.

## Literature review

### Approaches to post-disaster recovery

The literature related to post-disaster recovery approaches is very limited, because most studies are related to reconstruction. For instance, Carrasco, Ochiai and Okazaki (2016) listed five types of housing reconstruction approaches: (1) the cash approach is financial aid for housing repair and renovation without technical assistance; (2) owner-driven reconstruction, a conditional financial aid for house reconstruction provided directly to individuals supported by technical assistance and regulations; (3) community-driven reconstruction, a financial and/or material help routed through community organisations, which are encouraged to participate in decision-making and reconstruction management; (4) agency-driven reconstruction *in situ*, hiring of a contractor by the government or non-government entity to replace houses in their predisaster location; and (5) agency-driven reconstruction in the relocation site is similar to agency-driven reconstruction *in situ*, but new houses are being built on a new site (Carrasco et al. 2016).

On the other hand, Chang et al. (2010) discussed four resourcing activity approaches for post-disaster housing reconstruction as follows: (1) government-driven resourcing – governmental entities and other authorities are the primary drivers of resource availability; (2) donor-driven resourcing – donors primarily manage the resourcing efforts; (3) market-driven resourcing – the construction market has a major influence on the availability of resources including instruments, forces and rules; and (4) owner-driven – house owners are responsible for reconstructing their homes with limited external aid through self-maintenance (Chang et al. 2010).

Bilau, Witt and Lill 2015 observed that in the aftermath of the Gujarat earthquake in 2001, five different reconstruction approaches were used: (1) owner-driven, (2) subsidiary, (3) participatory, (4) contractor-driven *in situ* and (5) contractor-driven *ex nihilo* approaches (Bilau et al. 2015). Santiago et al. (2018) reduced the reconstruction and recovery approaches into two broad categories: (1) owner-driven, either at the community or individual level; and (2) agency-driven, in which reconstruction takes place at on- or off-site, for example, at the relocation site, and the agency concerned is a donor or an organisation implementing a project funded by other entities.

The agency-driven approach is considered the easiest and quickest way of returning to places affected by disaster to normality and protect communities with high numbers of vulnerable individuals. To ensure supply, quality and costs are controlled, the approach represents a balance of technical feasibility, building and planning regulations. Hence, it is called a ‘one-size-fits-all’ approach, which means not considering specific needs and diversities within the community (Elkahlout 2020). Compared to the owner-driven, the agency-driven approach proved to be undesirable for the beneficiaries, as it tends to suit donors and implementing agencies and seldom involves target community active participation. Moreover, serious attempts at understanding immediate demands or longer-term aspirations of households and communities, classes or cultures different from theirs are infrequent (Elkahlout 2020).

### The transition from owner-driven to community-driven approach

The owner-driven approach is based on the relevance of beneficiaries’ choices and various coping methods expected to be adopted to mitigate disaster impacts. Beneficiaries who participate in the aid design and construction process receive psychological healing impacts by being empowered and provided with a sense of control, and in the end, proper help is possibly received according to their needs. With the owner-driven approach, beneficiaries have a strong incentive to rebuild and restore their primary dwellings much faster and for less money, as opposed to donor-funded initiatives, which possess extended waiting periods of months or even years (Andrew et al. 2013; Lyons 2009; Ratnayake & Rameezdeen 2008).

The owner-driven approach is limited to household initiative and involvement in the recovery process. This is different from the systematic community-driven recovery, which is established by collective community-based organisations and not only limited to the household initiative. Furthermore, both approaches ought to be separated because they have varied decision makers. Based on the foregoing, the disaster recovery approaches are proposed to be divided into three broad categories: (1) community-driven, community-based, or community-led recovery – the community as a collective or its contained organisation majorly designs and manages the recovery with or without external support (Cretney 2018; Dhungana & Curato 2021; Nakanishi & Black 2016; Shaw 2014; Sullivan & Sagala 2020); (2) owner-driven recovery – individuals, groups or households as beneficiaries participate in the design and management of the recovery process; and (3) agency-driven recovery – the recovery process is managed and designed by the agency with slight participation from beneficiaries.

## Method

A qualitative research method involving focus group interviews (FGI), interviews and PRA was applied to explore and capture community narratives. Loli Dondo, as a fishermen’s village that received both agency- and community-driven livelihood recovery, was purposively selected for the case study. In 2019 and 2021, data were collected on community story experiences concerning the implementation of both recovery approaches using the same participants. Participatory rural appraisal, a family of methods including problem trees, Venn diagrams and management flows, was adopted to gather and analyse the data. This enables local people to share, enhance and analyse their knowledge of life and conditions, as well as to plan and act (Chambers 1994). Participatory rural appraisal is effective in producing valid and reliable data from local knowledge (Campbell 2001). It also involves participatory diagramming with other techniques such as interviews and observation (Sinthumule & Mudau 2019).

In total, 12 interviewing sessions and five focus group discussions were conducted for 20 participants with different repetitions for every individual, depending on their type and experience (see Table 1). A respondent-driven or snowball sampling technique was also used, where a participant facilitates the identification of additional participants and their selection.

**TABLE 1:** Number and type of participants.

Number	Type of parcipants	Number of parcipants	Year of interview
1	Head of fishermen group	1	2019 and 2021
2	Secretary of fishermen group	1	2019 and 2021
3	Member of fishermen group	13	2019 and 2021
4	Fishmonger	3	2019 and 2021
5	Mining laborer	2	2021

**-**	**Total**	**20**	-

This study aimed to collect past information (during the 2018 tsunami) on the community’s collective process during emergencies and its observation of agencies, as well as the way people fulfilled emergency needs. Also, present (including current conditions of the community after agency intervention) and future information on how people obtained useful and continuous help or aid after the agencies’ departure were to be obtained. These three were used to understand the community, post-disaster situations, changes occurring after intervention from agencies and how beneficiaries were impacted by the different recovery approaches, which would provide a comprehensive idea of community problems, potentials, advantages and solutions.

### Ethical considerations

Permission to conduct this research was obtained from Disaster Management Centre, University of Pembangunan Nasional Veteran Yogyakarta (reference number: No. 1/VI/LPPM-Etik/2022), local municipalities, chiefs and headmen. Informants were interviewed based on their willingness and availability. Respondents were assured that their answers would be kept confidential; to implement this, the names of interviewees are not revealed in this paper.

## Results and discussions

### Community self-recovery effort after the tsunami

**FIGURE 1:**
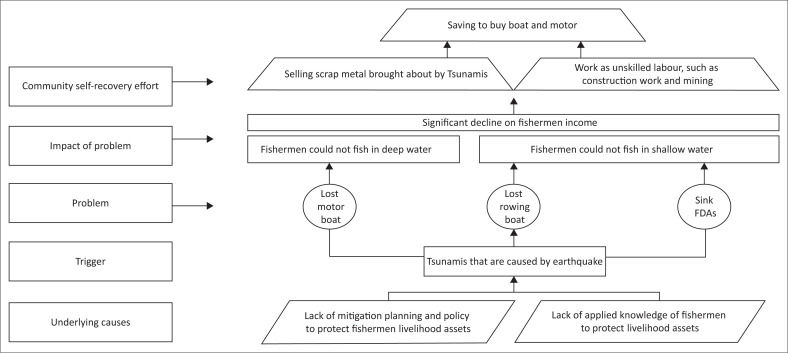
Problem tree mapping of Loli Dondo village fishermen.

Information was obtained through key informant interviews in 2019 with 12 participants from seven different households living in the nearest houses to the coastal area, while all of them are fishermen group members. The informants explained community situations experienced in implementing self-recovery efforts after the tsunami (see Figure 1). The earthquake occurred one evening after the fishermen had just returned home and their wives were busy assembling caught fish to sell:

‘That day, conditions were, as usual, my son was playing with the swimming buoy on the beach, and my wife was stringing fish. We didn’t feel anything strange would happen.’ (Participant 1, Male, Head of fishermen group)

After the earthquake occurred, coastal residents panicked and immediately evacuated to hills on the west coast. Geographically, the west coast area of Loli Dondo Village was favoured by the community near the hill. Therefore, the evacuation process was carried out faster in reaching a safe point:

‘We are very lucky here; the hill is close to the settlement. Even without disaster preparedness training, people here already understood that if there is a big earthquake there would be a tsunami; hence, they immediately ran to the hills. My wife and I ran to the hills, and so did other people. In the dim darkness, our houses were totally damaged and the rubble was swept away by the waves. Everyone was frantically looking for their family members. My son was lost but luckily survived.’ (Participant 3a, Male, Fisherman)

The next morning, residents saw their houses destroyed by the tsunami and tried to survive in the rubble and makeshift tents provided by the government and humanitarian agencies:

‘We have to be creative in these conditions. We rebuilt the monitoring post from the remains of fisheries construction. My house over there that has zinc cladding was built from what was left of my house, with the help of friends from our fisherman organisation. Living in a house with zinc cladding is better than in a tent. Living in a tent is difficult; when sunny it becomes very hot, and it floods during rains. There is no temporary shelter aid in our village from any organisation or government. It could be because the tsunami impact is not as severe as in Palu City.’ (Participant 1, Male, Head of fishermen group)

The residents’ means of livelihood, boats and traditional fishing aggregating devices (FADs) were lost in the tsunami. Fishing aggregating devices function by inviting large fish presence near the coast and are also useful specifically when the fishermen could not sail to the open water:

‘With the loss of the FADs, boats, and their motor, we obviously couldn’t do anything to fulfill daily needs. All boats were lost except for those (fishermen) fishing at the sea during the tsunami occurrence. We have a traditional FAD, which actually belongs to the next village, but we have to use it together because good relations exist between fishermen organisations. So we are allowed to fish on their FAD, as long as we don’t do netting. Because if we use netting method, all the small fish will be lifted without luring any big fish.’ (Participant 4, Female, Fishmonger)

The loss of fishermen’s production means causes loss of livelihood, hence their jobs change to unskilled labor, such as construction or mining-company workers. This situation was worsened by damaged coral reefs because of the tsunami, thereby disrupting the ecosystem and leaving no fish in shallow water:

‘With no income and aid, I took the initiative to rummage irons, metals and aluminum washed away from the port to the shore by the tsunami. Also, there was no company to take care of the containers. Therefore, I made sure scrap metal collectors want to buy our junk. When they said they could, we immediately searched for it on the coast with a tendency of earning 500 000 to 800 000 rupiah a day. Sometimes I took the door of the container that was drifted away and made a good amount of money with it. I just needed diving goggles and sacks to do this job. I had initiated this at first, and after, our neighbor started to do the same.’ (Participant 3b, Male, Fisherman)

‘Sometimes I worked as a construction worker, cleaner in a mining company and sometimes do scrap metal rummaging. One time while I was rummaging, I found a motorboat, which I tried to repair, and thanks to God it worked, so I could go back to the sea for fishing. I use that motor until now and it helps my family become better.’ (Participant 5a, Male, Mining laborer)

### Fishermen’s livelihood perception and pattern

The community selected fishermen’s livelihood, based not only on profit matters but also how inhabitants are provided with a decent life by the sea (see Table 2):

‘I could earn IDR500 000 to IDR800 000 ($34.50 to $55.00) just by three hours fishing and selling to the middleman. If the season is bad, my earnings could be at least IDR200 000 to IDR300 000 ($14 to $21) each day. Of course, it is less compared to when I worked as a mining machine operator, with a salary of IDR5 000 000 ($345) per month, including overtime. Being a fisherman is more profitable, even though the income is uncertain and depends on fish season. This means our earnings depend on luck and uncertainty, but for me, more time to spend with children and family is priceless.’ (Participant 5b, Male, Mining laborer)

‘We had 24 residents who worked as full-time fishermen, and in the past, some of us worked as part-time clove farmers. However, now, none of us work partly as a farmer, because farming today is quite a waste of time, as we need to go to the area over kilometers. Furthermore, the profit is getting lower and lower. During the dry season, farmers cannot plant any crop, and sometimes, they go fishing only when preferred.’ (Participant 1, Male, Head of fishermen group)

**TABLE 2:** Loli Dondo villagers’ livelihoods, income and problem analysis.

Livelihood	Actor		Income	Market	Problem
	Male	Female			
Fishing	V		IDR500 000 – IDR800 000 ($34.50 – $55.00)/3 h. In bad season, only IDR200 000 – IDR300 000 ($14 – $21)/day,	Middleman	Based on the fish season.
Traveling fish merchant		V	IDR150 000 – IDR250 000 ($10 – $17)/day	Housewives	Fewer buyers aer the tsunami.
Machine operator	V		IDR5 000 000 ($345)/month	Corporation	No me for the family.
Cleaning service	V	V	IDR50 000 – IDR100 000 ($3.5 – $7)/day	Corporation	Based on calls, not an everyday job.
Rummaging scrap metals	V	V	IDR500 000 – IDR800 000 ($34.50 – $55.00)/day	Scrap metal buyers	Scrap metal only exists a few weeks after the tsunami.

This condition makes Loli Dondo village fishermen choose to stay in their jobs, and they have no intention of changing it for another.

### Community perception of aid

Information was obtained through an FGI in 2019 with 15 participants registered under the fishermen organisation, who mostly lost their jobs after the tsunami. Focus group interviews was implemented to show that all aid is supposed to fulfil fishermen’s needs and standards. In case the aid is only provided partially, it tends to be unable to help the beneficiaries. In making a living, fishermen have a combination of tools; for example, motor or rowing boats cannot be used unless additional engines or FADs are offered as one unit of aid. Because of the FGI, fishermen identified the following three important aspects fundamental to the continuity of fishing activity (see Table 3):

1. Fishermen with motorboats have a strong dependency on engines, because their size makes it impossible to row to catch fish. These people do not need FADs because they fish at points far from the bay, where the big fish are active.
2. Fishermen with rowboats need a definite fishing spot on FADs near the coast, because rowing boats are not used for deepwater fishing. Additionally, FADs lure big fish into the shallows to promote the possibility of being captured.
3. The rowboat size is different once compared to the motorboat and is normally 5 m – 6 m with a width of 60 cm. Meanwhile, the motorboat size ranges from 7 m to 8 m, with a width of 60 cm – 90 cm, and is a community standard. Certainly, this cannot be changed due to the tendency of affecting the boat’s function and effectiveness.

**TABLE 3:** Loli Dondo fishermen organisation’s priority ranking on the forms of aid.

Forms of aid	Rank	Explanation
Motorboat (with engine)	1	Motorboat makes it possible to fish in deep water areas that have a high value and are the best way to improve income.
Motor	2	The motor is expensive and hard to get, but the boat can be bought in secondhand form from other fishermen.
Rowboat	3	Even without FAD, using a rowboat for fishing in shallow water is possible; however, it tends to be hard.
Fishing aggregating device	4	A FAD is an investment for the future, and it is possible to buy the rowboat later with savings.
Motorboat (without motor)	5	The motor is expensive and hard to get since its vendors only exist in the city; besides, rowing a motorboat is really hard.
Fishing gears	6	Fishing gears are cheap and easy to access, but they can be borrowed from other fishermen.

FAD, fishing aggregating devices.

**FIGURE 2:**
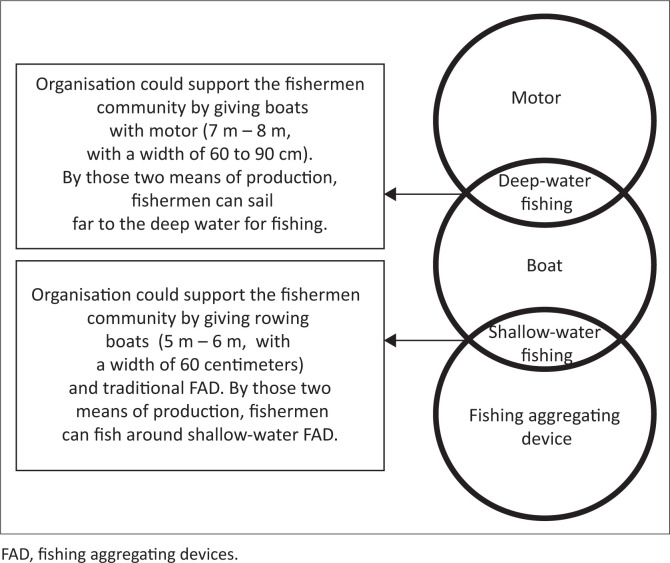
Venn diagram of best recovery aid for fishermen.

The three components, boat, motor and FAD, are essential and must be understood from the needs of the fishing community members in carrying out their work. Based on the PRA approach, the Venn diagram of the Loli Dondo fishermen’s organisation seeks a further explanation of the relationship between each component (see Figure 2).

This Venn diagram emphasises the fishermen’s consensus that their condition is not recoverable provided the support only fulfills one of the three items:

‘It is useless if we receive only boats without motor, or rowing boats without FADs. But it is better if we receive rowing boats only; at least we could capture small fish. However, to improve our family economy will be really hard.’ (Participant 1, Male, Head of fishermen group)

This basic proposition was not implemented by agencies that came to the village. The community saw that aid distribution had a huge discrepancy. This was shown by the situation where a village had accepted many boats, while there was a neighbouring village yet to receive any help, even though both had experienced similar damages:

‘Until now, aid has not been evenly distributed in coastal villages in Donggala Regency. Some received many boats, but others did not receive any assistance at all.’ (Participant 1, Male, Head of fishermen group)

‘We only saw boats being transported by trucks, given to the neighboring village, but nothing for ours. The villages that had not received aid are Loli Dondo and Loli Pesua. Meanwhile, Loli Tasiburi and Loli Saluran villages already received boats and motors from the Floating Hospital and other organisations. Often, people disappointed us, particularly those who only came to collect data, while the aid was provided to other villages.’ (Participant 2, Male, Secretary of fishermen group)

Donggala District Office was unable to support the community through the insurance scheme. The fishermen’s group is part of the fishermen insurance scheme managed by the Donggala Regency government. However, the risk of losing boats is not covered by insurance, and thus the insurance payouts cannot be claimed:

‘The government did not give us the right to claim the fishermen’s insurance program, even though we always paid the charges annually. We understand that the insurance does not have a clause of “if the boat is lost”, rather is only about the loss of life, disability, or injury. But the government should have some empathy for us because this is a tragedy.’ (Participant 1, Male, Head of fishermen group)

### Agency-driven recovery practice

**FIGURE 3:**
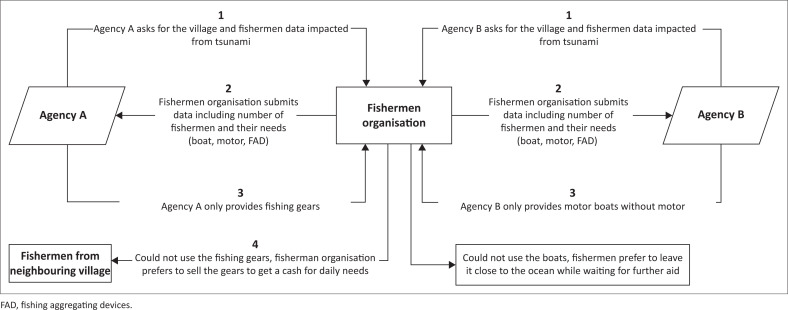
Management flow diagram on the practice of agency-driven recovery by Agencies A and B.

**FIGURE 4:**
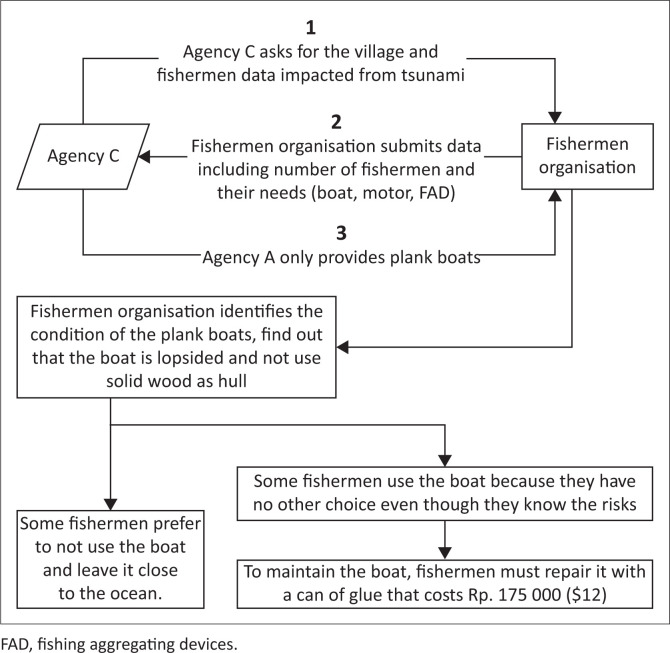
Management flow diagram on the practice of agency-driven recovery by Agency C.

Information was obtained through FGI in 2019 with 15 participants who received help from all agencies and were registered under the fishermen organisation. In implementing agency-driven livelihood recovery in Loli Dondo village, Agencies A, B and C involved the community in seeking the beneficiaries’ data and needs, but not fully in the realisation process, including obstacles present. Consequently, even though correct data were provided, the aid items did not meet the community’s criteria and they became useless. This condition was highlighted through FGI with the PRA approach of a management flow diagram to find a communication gap between the community and donor agencies as well as Non-Governmental Organizations (NGOs), both local and international (see Figure 3 and 4):

‘Certain organisation [*Agency A*] had donated fishing gears, but in the end, we sold it because of the uselessness. We did not have a boat to sail and use the gears.’ (Participant 3c, Male, Fisherman)

‘The boats provided by the NGOs that came to Loli did not meet our standards. Hence, they were not considered to be an aid by the fishermen. In the end, we only placed the boats on the shore. One organisation [*Agency B*] provided motorboats but without a motor, thereby leading to not being used by the fishermen. It would be better if we received motor only or rowing boat instead.’(Participant 2, Male, Secretary of fishermen group)

‘Another organisation [*Agency C*] provided boats that did not meet the fishermen’s community standard. Three weeks after the tsunami, a group of foreigners arrived to collect data and promised the provision of boats to all fishermen. But in the end, what they gave to us was this lopsided boat. You can see that the boat only uses planks, without using solid wood for its hull. This is dangerous based on the lopsidedness because it will become unstable when hit by the waves.’ (Participant 1, Male, Head of fishermen group)

‘Firstly I tried to use that lopsided boat. But because the composition is made of planks only, it is always broken due to waves and engine vibrations. To keep the boat intact, I must repair it with a can of glue that costs IDR175 000 ($12). A can of glue is used for one-time repairing only and several sailing times until it becomes damaged again and needs to be repaired. If I go further into the sea, more severe damage will occur. If sailing is performed without repairing this boat, I will surely die from drowning. Hence, I stopped using the boat due to high maintenance cost.’ (Participant 3b, Male, Fisherman)

Community members already emphasised the importance of solid wood for the boat’s hull. They agreed that the boat ought to be built with solid wood, not planks:

‘We had provided fishermen organisation data, the price of engine boats and the importance of solid wood for boat’s hull. They disrespected us; in the end, the boat was wasted, and this disappointed the community after waiting so long and expecting the aid.’ (Participant 2, Male, Secretary of fishermen group)

### Community-driven recovery practice

**FIGURE 5:**
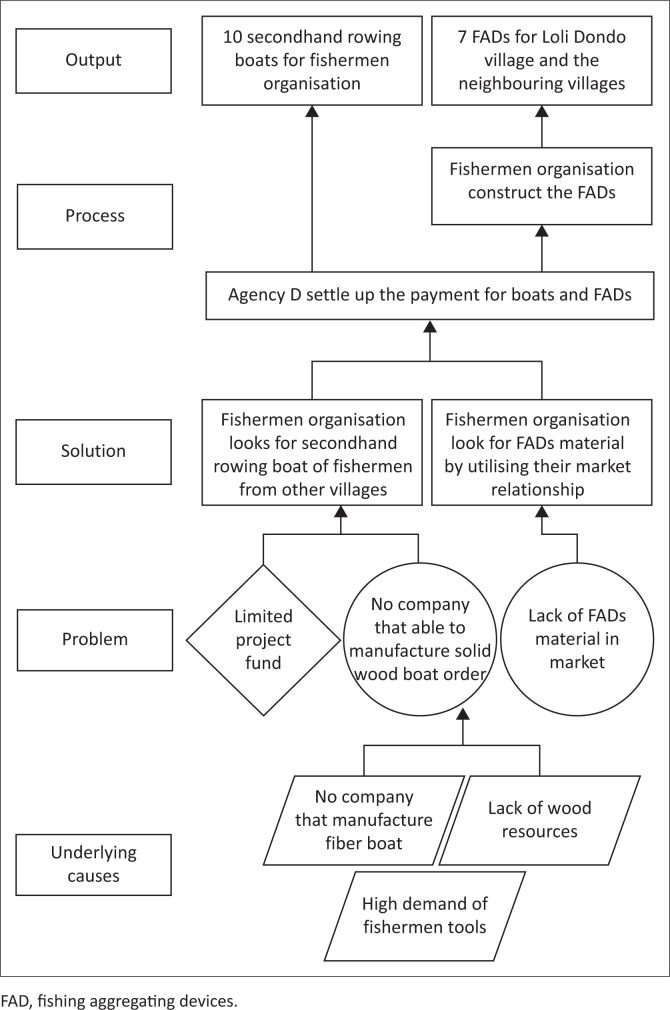
Community reflection on the comparison of agency-driven and community-driven approaches.

Information was obtained through an FGI performed in 2019 with 15 participants who are registered under the fishermen organisation and involved in the process of support provided by Agencies A, B and C. In implementing community-driven livelihood recovery in Loli Dondo village, Agency D involved the community to seek beneficiaries’ data and needs, as well as implement the plan and control the quality of items that they will accept (see Table 4). Community participation used by Agency D helped to determine the problem-solving strategy lacked by Agencies A, B and C, hence the community supports the project’s realisation (see Figure 5):

‘When they [*Agency D*] came to us, they presented themselves as a researcher, seeking data on boat distribution in the coastal village of Donggala Regency. We never knew they already collected preliminary data to distribute boats too, because they had never promised us anything. Some questions were only related to how the boat would be distributed and villages that had never received any boats before. Shortly after this, the delivery of their plan to provide aid for our fishermen’s organisation commenced.’ (Participant 1, Male, Head of fishermen group)

‘They held a one-time focus group discussion on the boat seeking process, field condition, and the fact that no more boatwrights in Donggala Regency were capable of making any boat back then, every boatwright was fully booked. We said the same thing as what we told the previous agency who had come to us, that we are working for ourselves and no aid has helped us effectively. But they claimed not to have huge funds; hence, we [*fishermen’s organisation*] were asked to find the best way to improve fishermen’s condition. On the same day, we decided to look for secondhand rowing boats and FAD materials because Agency D’s budget was not enough for motorboats.’ (Participant 2, Male, Secretary of fishermen group)

‘We decided to look for secondhand rowing boats. After finding the boats, we informed Agency D, and money was transferred to us to buy the boats. The payment method for FAD materials was also the same. After obtaining the materials, we did communal work to build the FADs which were put in the sea. Then, we sent the photos to Agency D as evidence that we had conducted our task distributed in the FGI. In the end, we received 10 rowing boats and 7 FADs. We had the responsibility to share the FADs with other neighboring villages for usage also.’ (Participant 2, Male, Secretary of fishermen group)

‘Due to boats’ limitation, the fishermen organisation made a “boat schedule” to ensure that every fisherman access the boats fairly, and they are not privately owned. We shared the 10 boats with 15 fishermen and in the end, we decided that every fisherman has 6 hours to use the boat daily.’ (Participant 2, Male, Secretary of fishermen group)

**TABLE 4:** Actions and outputs from community-driven recovery practices.

Number	Actions	Outputs
1.	Agency D came to Loli Dondo village to perform a baseline study.	Baseline data
2.	The fishermen’s organisation provided data and conditions of Loli Dondo village.	Community’s data
3.	Agency D returned to explain the project idea and held FGI with the fishermen’s organisation to plan the project’s realisation.	Problem and solution analysis
4.	The fishermen’s organisation agreed to realise the planning.	Oral agreement
5.	The fishermen organisation searched for secondhand rowing boats and FAD materials from their networking of fishermen in other villages.	Informaon about the sellers and price
6.	Fishermen’s organisations purchased the rowing boats and FAD materials with the project funds	Receiving of boats and FAD materials
7.	Agency D and the fishermen’s organisation communicated through instant messaging applications to elaborate on the progress, report and solution to the project problems.	Reporting through instant messaging applications
8.	Due to the boats’ limitaon, the fishermen’s organisation made a ‘boat sched	Boat usage schedule
9.	Beneficiaries used the boats and FADs.	Community income

FAD, fishing aggregating devices.

**FIGURE 6:**
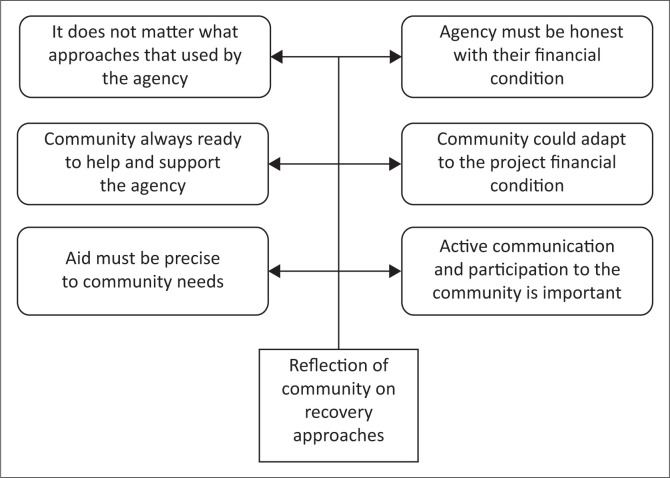
Community reflection on the recovery approaches in Loli Dondo Village.

The community declared being available in case any agency needs help in realising the programme. Furthermore, sentences mentioned frequently in the group interview were ‘to help the agency’ and ‘to realise our own needs’. The community described the aid items’ precision as the most important, followed by their speed of arrival (see Figure 6). Therefore, in any form of the approach, with or without the participation of beneficiaries in early recovery, fishermen need aid items as exactly stated by the community:

‘To be honest, I prefer to be helped quickly. Hence, when the agency came and gave the appropriate support needed, it was what we hoped for, although such has never happened before. The agency that came and communicated with us except the last one (Agency D), did not really understand our needs.’ (Participant 3d, Male, Fisherman)

‘I think the last NGO implemented a good strategy, by knowing their weakness on the fund, they talked to us and asked for our support. We gladly helped, because in the end, we were the ones who benefited from the help.’ (Participant 3e, Male, Fisherman)

‘In the end, I agreed that the aid earliness was nothing if it was not suitable to our needs.’ (Participant 3d, Male, Fisherman)

‘I really enjoyed the process of helping with the Agency D aid realisation. It has been very long since we worked together to achieve our common goals and build FDAs, so our community could earn better. Communication with the fishermen from the neighboring village also makes us aware of other conditions.’ (Participant 1, Male, Head of fishermen group)

‘As previously stated, we, as beneficiaries, just need rowing boats or FDAs. However, external organisations need to be honest; if they are unable to fulfill our wishes, we could adapt and help too.’ (Participant 2, Male, Secretary of fishermen group)

### Community-driven recovery impact on community livelihood

**FIGURE 7:**
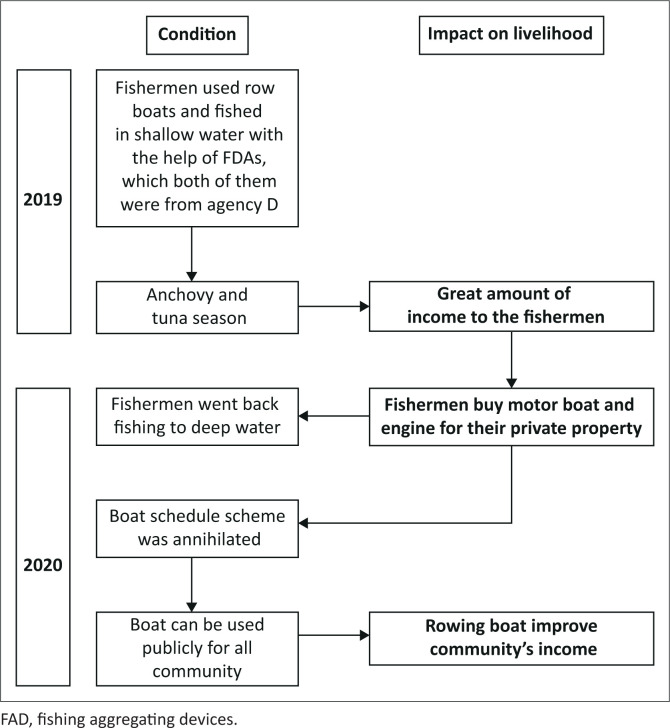
The impact on livelihood 2 years after the community-driven project.

Information was obtained through three FGIs in 2021 with 17 participants who accepted aid from all agencies and are registered under the fishermen organisation, plus mining labourers who also benefited from the aid. Additionally, it was implemented to determine the changes from 2019 to 2021, including which agency proved to be the most influential towards the community within these years. The result showed how Agency D performed the community-driven approach process effectively, and also impacted the fishermen group well (see Figure 7). This was indicated by the increasing income and life quality of the community and fishermen in the last 2 years:

‘In recovering our economy, at first, we used boats that we obtained together with Agency D. In 2019, these boats led us to the most essential fish season, in which in 2020, we were able to buy motorboats and the engine.’ (Participant 3e, Male, Fisherman)

‘The fishermen, at that time, reaped well and our welfare was increased. There were two important fish seasons, including anchovies and tunas, which certainly helped us. A great season in 2020 was really useful for us to increase our welfare, and to buy motorboats and the engine. Afterward, we took advantage of the big income in 2021 to renovate our house and save our money.’ (Participant 2, Male, Secretary of fishermen group)

‘There were three FADs provided by Agency D. Two are still there and one of the three is wrecked as it was hit by a barge, but normally, a FAD can last for 5 years.’ (Participant 3d, Male, Fisherman)

‘Boat management was also changed as fishermen possessed their own boats. The “boat schedule” scheme was changed into public-owned, as the boats were placed on the beach edge and used by interested fishermen. To maintain their continuity, we took care of the boats periodically. Nowadays, it is a fact that laborers often use the boat to earn extra income by shallow water fishing.’ (Participant 1, Male, Head of fishermen group)

## Conclusion

The analysis results showed that the agency-driven approach failed to meet the community’s appropriate needs, despite stating the members’ data and needs. Although a longer time is required, the community-driven approach enables the community to adapt and manage the projects to solve existing problems and actualise their aid perfectly. This seems to be in line with Elkahlout (2020) who also described it as desirable for beneficiaries because of the active participation in the aid process.

Moreover, PRA methods show the mapping of needs, problem-solving strategy visualisation and management patterns employed by the community as follows: (1) problem tree mapping is used for benchmarking similar problems in fishermen’s communities affected by tsunamis in other areas; (2) villagers’ livelihood income and problem analysis tend to be used as an alternative for low-income families; and (3) Venn diagram of best recovery aid and priority ranking on forms of aid are used for benchmarking the most appropriate aid for fishermen.

The community participative process supported the community-driven project realisation; therefore, it tends to overcome all problems that emerged. For example, the absence of boatwrights to produce solid wood boats and limited project funds were solved after the fishermen’s organisation was involved in search of secondhand boats from other fishermen in neighbouring villages. A collective network of fishermen’s organisations could also drive FADs provision, which happened to be scarcely provided after tsunami.

Hence, three key elements of recovery implementation were identified based on the community perspectives as follows: (1) transparency of the agency to the community about financial conditions; (2) active communication and involvement of the community; and (3) preciseness of the aid to community needs.

The problem-solving process could be implemented because the strategy jointly created by the community was proved to be adaptive, simple and effective. Community skills on relevant knowledge and field situations serve as important assets in process planning, and they are something that the agency does not possess. This study also highlights the lack of transparency process to ensure the quality control of aid by both agency and community. Therefore, the monitoring and evaluation mechanism in disaster assistance must be encouraged through regulations and guidelines, including the involvement of the National or Regional Disaster Management Agency as an institution that has full authority in disaster management.
